# OCMA: Fast, Memory-Efficient Factorization of Prohibitively Large Relationship Matrices

**DOI:** 10.1534/g3.118.200908

**Published:** 2018-11-27

**Authors:** Zhi Xiong, Qingrun Zhang, Alexander Platt, Wenyuan Liao, Xinghua Shi, Gustavo de los Campos, Quan Long

**Affiliations:** * Department of Computer Science, Shantou University, China; † Department of Biochemistry and Molecular Biology, University of Calgary, Canada; ‡ Annie Charbonneau Cancer Institute, University of Calgary, Canada; § Center for Computational Genetics and Genomics, Temple University, USA; ** Department of Mathematics and Statistics, University of Calgary, Canada; †† Department of Bioinformatics and Genomics, University of North Carolina at Charlotte, USA; ‡‡ Department of Epidemiology & Biostatistics, Statistics & Probability and Institute for Quantitative Health Science and Engineering, Michigan State University, USA; §§ Department of Medical Genetics, University of Calgary, Canada; *** Alberta Childrens Hospital Research Institute, University of Calgary, Canada

**Keywords:** Eigen decomposition, Singular value decomposition, Genetic matrices, Memory virtualization, Gene mapping, Genotype-based phenotype prediction, Genomic selection

## Abstract

Matrices representing genetic relatedness among individuals (*i.e.*, Genomic Relationship Matrices, GRMs) play a central role in genetic analysis. The eigen-decomposition of GRMs (or its alternative that generates fewer top singular values using genotype matrices) is a necessary step for many analyses including estimation of SNP-heritability, Principal Component Analysis (PCA), and genomic prediction. However, the GRMs and genotype matrices provided by modern biobanks are too large to be stored in active memory. To accommodate the current and future “bigger-data”, we develop a disk-based tool, Out-of-Core Matrices Analyzer (OCMA), using state-of-the-art computational techniques that can nimbly perform eigen and Singular Value Decomposition (SVD) analyses. By integrating memory mapping (mmap) and the latest matrix factorization libraries, our tool is fast and memory-efficient. To demonstrate the impressive performance of OCMA, we test it on a personal computer. For full eigen-decomposition, it solves an ordinary GRM (*N* = 10,000) in 55 sec. For SVD, a commonly used faster alternative of full eigen-decomposition in genomic analyses, OCMA solves the top 200 singular values (SVs) in half an hour, top 2,000 SVs in 0.95 hr, and all 5,000 SVs in 1.77 hr based on a very large genotype matrix (*N* = 1,000,000, *M* = 5,000) on the same personal computer. OCMA also supports multi-threading when running in a desktop or HPC cluster. Our OCMA tool can thus alleviate the computing bottleneck of classical analyses on large genomic matrices, and make it possible to scale up current and emerging analytical methods to big genomics data using lightweight computing resources.

Supported by recent advancements of high-throughput sequencing technology, several organizations have developed very large biobanks comprising DNA sequence linked to phenotypic and demographic data, *e.g.*, Genomics England The 100,000 Genomes Project (Peplow 2016). Some of these resources have been shared to worldwide scientists and practitioners (Collins 2012). This abundance of data brings unprecedented opportunities to develop a broad range of applications in precision medicine such as predicting drug response and disease risk for new patients. The same trend is evident in agriculture, especially with the recent trend of “high-throughput phenotyping” that generates high dimensional phenotypes. However, on the way to enacting this vision, a pressing roadblock is that the datasets are typically too large to be analyzed in the main memory of many existing computing infrastructure, including some High-Performance Computing (HPC) clusters or Cloud computing. As such, effective use of these biobanks is being relegated to the use of HPCs or computing nodes equipped with huge amount of memory (at the level of terabytes). To pave the path to the routine use of precision genomics in all clinics in the near future, it is preferable to carry out most statistical inferences of large genomic matrices involving hundreds of thousands individuals on personal computers, HPC or Cloud computing nodes of limited memory within tens of GB.

Previously, we have developed JAWAmix5, a hard disk-based solution to large data problems to allow for quick analyses of biobank-sized genotype data on computers with limited memory (Long *et al.* 2013). JAWAmix5 offers scalable functions for genotype-phenotype association mappings by storing the genotype data in disk using HDF5-based data structure. However, the continued growth of biobanks has given rise to new challenges that require further development and extension of these memory-efficient approaches. Now, in addition to large amounts of genomic data, the matrix that represents pair-wise genetic similarities between all the participants in a cohort of a biobank (the Genetic Relationship Matrix, or GRM) may require tens of billions of entries, becoming as sizable as the genotype data itself. These GRMs often play central roles in genomic analyses (Speed and Balding 2015; Kim *et al.* 2017). In particular, the eigen-decomposition of GRMs is the most time-consuming step that is required in many routine analyses such as Principal Component Analysis (PCA), a default model for high-dimensional data visualization (Ringnér 2008) and gene expression analyses (Stegle *et al.* 2012); estimation of genomic-heritability using seemingly unrelated subjects (Yang *et al.* 2011; de los Campos *et al.* 2015), a first step to understand the genetic architecture of a trait; linear mixed models (LMM) (Kang *et al.* 2010), a popular approach in genotype-phenotype association mapping; Genomic Best Linear Unbiased Predictors (Clark and van der Werf 2013), and phenotype predictions (de los Campos *et al.* 2013; Pérez and de los Campos 2014). Although some of the methods such as SNP-BLUP can avoid explicitly factorizing a GRM (Koivula *et al.* 2012), factorizing a matrix is still a main technique needed by most analytic models.

The frequently used matrix-factorization as an alternative to eigen-decomposition is the Singular Value Decomposition (SVD) of a rectangular matrix (*e.g.*, a genotype matrix) which avoids heavy computations but approximately achieves similar precision. For instance, in the setting of a LMM, David Heckerman’s group has developed a method that selects only a subset of genomic variants to form a genotype low-rank matrix that can be solved faster and lead to fewer false positives (Lippert *et al.* 2011; Listgarten *et al.* 2012; 2013). Many tools have soon adopted this approach as an alternative of solving eigen-decomposition of a full GRM.

Additionally, many emerging novel analytical procedures, such as a recent proposal for correcting the confounding effects of cell-type compositions (Rahmani *et al.* 2016) in epigenome-wide association studies and the correction of confounding effects in the analysis of single-cell RNA-Seq data (Buettner *et al.* 2015), need factorization of large matrices.

Researchers have also resorted to approximations to evade the computational burden of calculating very large matrices. For PCA analyses, Alkes Price’s group developed an approximate algorithm using only the top few PCAs (Galinsky *et al.* 2016). For calculating the inverse of a GRM of many related animals, one may start with “Core Animals” to approximately solve the whole matrix (Masuda *et al.* 2016). In the R statistical programming language community, chunking algorithms and memory-mapping techniques are utilized to reduce the memory use for simple functions such as reading data and conducting linear regression (*e.g.*, The Bigmemory Project, http://www.bigmemory.org). Parallelization strategies using “split-apply-combine” have also been implemented such as in the BGData suite of packages (de los Campos and Grueneberg 2017). There are many efforts using Graphics Processing Units (GPUs) to speed up computations, for instance BLASX (Wang *et al.* 2016) for numerical computations. Additionally, some researchers used distributed memory to solve the problem of limited memory on a single server, such as Elemental (Poulson *et al.* 2013). Although these approaches address individual problems on a case-by-case basis, researchers need universal and exact solutions for eigen-decomposition as well as singular value decomposition of very large GRMs and genotype matrices for various existing and future applications.

The above applications are evidences that the eigen-decomposition (and its alternative, SVD) in genomic studies is indeed the first-line characterization of the relationship between participating individuals and the solution implementing these on ubiquitously available computer hardware is needed. In this work, we present a novel tool, OCMA, to factorize very large matrices using disk-based solutions. This tool, in conjunction with the other tools developed by us (Long *et al.* 2013) and others, will allow researcher to carry out association mapping and phenotype predictions nimbly using limited computational resources.

In computer science, “*mmap*” (Figure 1) is a function (or, precisely, a “system-call”) that maps files or devices into memory (precisely, maps to the virtual address space of a computing process). “*mmap*” was originally designed as part of Berkeley Software Distribution version of Unix (McKusick *et al.* 2014) and is now universally supported by all major operating systems (*i.e.*, Mac OS, Windows and Linux). This function efficiently handles data exchange between memory and disk, allowing program developers to carry out computational tasks as though the data in the disk were in memory without taking care of implementation details such as allocating memory, looking up addresses, reading data, writing used data, and de-allocating the memory again when it is no longer needed (Song *et al.* 2016). As a result, it appears that the calculations happen “out of the memory”, a technique called “*out-of-core*” in computer science. Mmap has been extensively used in many computing fields such as high-performance computing (Van Essen *et al.* 2013; Song *et al.* 2016), graph computation (Lin *et al.* 2014), system virtual machine (Wang *et al.* 2015), and databases (Lou and Ludewigt 2015). Mmap has also been used in bioinformatics in recent years, including the indexing of next-generating sequencing reads (Salavert *et al.* 2016) and tree-based backward search (Salavert *et al.* 2015).

**Figure 1:**
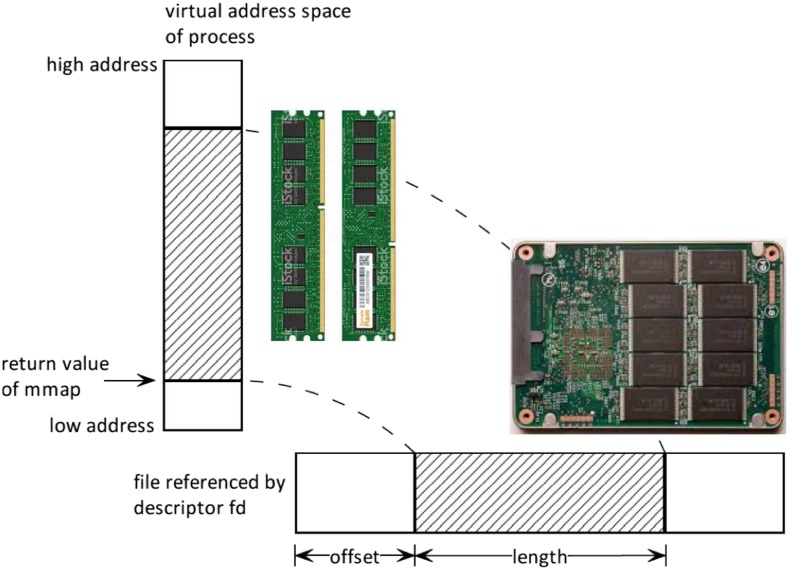
Illustrating schema of *mmap*. The horizontal bar represents the physical address in the disk and the vertical bar represents the virtual address from the perspective of a computing process in the memory.

## Methods

The *Intel Math Kernel Library* (Wang *et al.* 2014) is a collection of optimized functions that represent the state-of-the-art for numerical computation. Using the core algorithm supplied by the Intel MKL and the memory-disk mapping enabled by mmap, here, we develop a tool called Out-of-Core Matrices Analyzer (OCMA) that solves eigen-decomposition of GRMs and SVD in the disk (*i.e.*, out-of-core).

In order to integrate Intel MKL with mmap, we have thoroughly assessed various options of implementation to optimize the performance of OCMA. In the current version, OCMA adopts the ILP64 library that needs the support of 64-bit machines to accommodate very large indexes of matrices. We have selected the “ssyevd” subroutine that computes all eigenvalues and, optionally, all eigenvectors of a real symmetric matrix, using divide and conquer algorithm. We have re-engineered the interface of “ssyevd” so that the large matrices used in it can be disk-based using mmap (Supplementary Note I). Similarly, we have identified “sgesdd” that computes the SVD of a general rectangular matrix using a divide and conquer method. Additionally, to meet many applications that one only needs a small number of singular values and eigenvectors, we have also implemented a function that calculates the top *k* singular-values (*k* < min (*N, M*) where *N* and *M* are the dimensions of a matrix, typically standing for the number of individuals and the number of genetic markers in a genotype matrix). This function is supported by the “sgesvdx” in Intel MKL. These subroutines require more workspace but are faster in some cases, especially for large matrices. Intel MKL provides a two-level C interface to LAPACK, consisting of a high-level interface and a middle-level interface. The high-level interface handles all workspace memory allocation internally, while the middle-level interface requires the user to provide workspace arrays as in the original FORTRAN interface. To facilitate mmap-based solutions, we have used the middle-level interface that is similar to its original FORTRAN interface. The details of the implementation are presented in Supplementary Note I.

OCMA supports parallel computing. By default, OCMA will automatically decide how many threads to use based on the available hardware. (In our tests to be presented in Results, the OCMA process uses four threads, which is based on the hardware of the desktop computer we use.) Additionally, the users can also utilize environment variable (MKL_NUM_THREADS) to specify the number of threads (OCMA Users’ Manual).

### Data Availability

We used the UK Biobank data that is available through application to the data generator (https://www.ukbiobank.ac.uk). Supplemental material available at Figshare: https://doi.org/10.25387/g3.7384973.

## Results

OCMA provides two main functions: eigen-decomposition for square symmetric matrices (*e.g.*, GRMs) and singular value decomposition (SVD) for rectangular matrices (*e.g.*, genotype matrices). The users can also select to calculate a subset of top singular values/vectors. The input files can be prepared using plain text or the mainstream binary format (detailed in the Users Manual). OCMA also supports the format transformation between text and binary files. The output files will be the eigenvalues/vectors (or singular values/vectors) in the same format as the input. All three operating systems (Linux, Mac OS, and Windows) are fully supported.

An example command using OCMA may be:

$> ocma eigen single disk n A E Q

where ‘**ocma**’ is the name of the executable; ‘**eigen**’ stands for eigen-decomposition (alternatively one may use ‘singular’ for SVD); ‘**single**’ stands for single-precision (alternatively one may use ‘double’ for double precision); ‘**disk**’ specifies the function that uses disk-based solution (alternatively one may use ‘memory’ for smaller matrices); ‘**n**’ is the dimension of the matrix; ‘**A**’ is the filename of the input matrix; ‘**E**’ is the filename of the output file that stores the eigenvalues of matrix A; and ‘**Q**’ is the filename of the output file that stores the eigenvectors of matrix A.

We first compared the results of an eigenvalue decomposition and an SVD computed using OCMA with the same factorization carried out using MATLAB functions “eig” and “svd” (version 2012b) (Table 1). As expected, the outcome of OCMA and MATLAB is highly consistent as quantified by the three standard distances (that is called *norm*) used in numerical computing (Table 1). Additionally, these initial comparisons also show that, for relatively small matrices that MATLAB can handle, OCMA is 4 – 11 times faster than MATLAB for the computation of eigen-decomposition and around 2 – 3 times faster than MATLAB for the SVD (Table 1).

**Table 1:** The algorithm of OCMA calculates the right outcome. Eigenvectors (or singular vectors) are compared using Infinite norm $\left({\left\|\text{*}\right\|}_{\infty }=\text{max}\{|x_{1}|,...,|x_{n}|\}\right)$, Taxicab norm $\left({\left\|\text{*}\right\|}_{1}=\overset{n}{\underset{i=1}{\sum }}|x_{i}|\right)$, and Euclidean norm $({\left\|\text{*}\right\|}_{2}=\sqrt{\overset{n}{\underset{i=1}{\sum }}x_{i}{}^{2}})$. We use $\lambda_{ocma}$ and $S_{ocma}$ to denote the vectors calculated by OCMA and $\lambda_{matlab}$ and $S_{matlab}$ to denote the vectors calculated by MATLAB (version 2012b). The comparison for eigen-decomposition is presented in the upper table, and the comparison for singular-value decomposition is presented in the lower one. In the upper table, *N* denotes the number of individuals. In lower table, *N* = 1,000,000, and *M* denotes the number of genetic markers. The configuration of the personal computer: Intel Core i7-6700 CPU (4 cores), Memory = 24GB. Disk = Samsung SSD 850 EVO 250GB. The operating system is Windows 7. Time is measured by wall-clock (instead of CPU time)

*N*	$\left\|\lambda_{ocma}-\lambda_{matlab}\right\|/\left\|\lambda_{matlab}\right\|$			Computation time (s)	
	${\left\|\text{*}\right\|}_{\infty }$	${\left\|\text{*}\right\|}_{1}$	${\left\|\text{*}\right\|}_{2}$	OCMA	MATLAB
1000	5.2*10^−7^	7.6*10^−8^	1.1*10^−7^	0.1	0.4
2000	2.8*10^−7^	8.1*10^−8^	1.0*10^−7^	0.5	2.8
5000	4.3*10^−7^	2.1*10^−7^	2.4*10^−7^	6.4	35.5
10000	1.6*10^−6^	5.7*10^−7^	6.3*10^−7^	46.2	294.0
20000	3.3*10^−6^	4.4*10^−6^	4.3*10^−6^	246.4	2905.1

*M*	$\left\|s_{ocma}-s_{matlab}\right\|/\left\|s_{matlab}\right\|$			Computation time (s)	
	${\left\|\text{*}\right\|}_{\infty }$	${\left\|\text{*}\right\|}_{1}$	${\left\|\text{*}\right\|}_{2}$	OCMA	MATLAB
100	2.9*10^−5^	1.2*10^−4^	8.9*10^−5^	10.2	12.3
200	2.1*10^−5^	9.2*10^−5^	6.6*10^−5^	14.8	29.6
500	1.9*10^−5^	7.7*10^−5^	5.6*10^−5^	47.5	90.4
1000	1.6*10^−5^	1.3*10^−4^	8.5*10^−5^	89.5	206.4
2000	1.2*10^−5^	1.1*10^−4^	7.1*10^−5^	226.1	726.9

To assess memory usage and computational time for analyses involving genomic big-data we used GRM describing additive genetic relationships among 100,000 distantly related (GRM between pairs of individuals smaller than 0.03) from the interim release of the UK-Biobank. This GRM was computed using 589,028 SNP genotypes from the Affymetrix UK BiLEVE Axiom and Affymetrix UK Biobank Axiom arrays (Collins 2012). The GRM was computed using the BGData R-package (de los Campos and Grueneberg 2017). Further details about the criteria used for subject and SNP inclusion can be found in (Kim *et al.* 2017) and the procedure to compute GRM is in Supplementary Note III.

When the GRM is small enough to be fully loaded in memory, we can directly compare the disk-based solution to the memory-based one (Table 2, *N* = 10,000 to 50,000). In most cases, the overhead incurred by disk-memory mapping only causes a modest slow-down in the disk-based solution compared to the memory-based one. Notably, taking advantage of the efficient implementation offered by Intel MKL, OCMA can decompose a regular matrix of *N* = 10,000 in less than a minute. More importantly, OCMA rapidly solves much larger GRMs that are impossible to load in memory (Table 2, *N* = 60,000 to 100,000). In particular, it solves a GRM of 100,000×100,000 in only 5 days in our personal computer that has only a memory of 24GB (Supplementary Table S4).

**Table 2:** Runtime and memory consumption of OCMA in a Linux personal computer for eigen decomposition. The same hardware in Table 1 is used. Operating system is CentOS Linux release 7.3.1611. Four threads are used for the test. The three columns in the “Calculation time” stand for the runtime for three tools: GCTA (Yang *et al.* 2011), OCMA using memory only, and OCMA using disk (based on mmap technique). The careful interpretation of the comparison between GCTA and OCMA is explained in the main text and Supplementary Notes II. *The usage is slightly larger than the physical memory because of the swapping by the operating system. **GCTA was tested in an HPC cluster when the memory of a personal computer is insufficient. Time is measured by wall-clock (instead of CPU time). Memory consumption is estimated using the formula on the GCTA website and the Intel MKL specification (detailed in the Supplementary Notes). The sign “/” indicates that the system does not allow the calculation to happen due to limited memory and swap spaces or it exceeds the maximal runtime in the HPC

*N*	Computation time			Memory usage	
	GCTA (GREML)	OCMA (Memory)	OCMA (Disk)	GCTA	OCMA (Memory)
10000	286.0 s	41.6 s	55.0 s	1.6 GB	1.1 GB
20000	2988 s	214.5 s	231.8 s	6.4 GB	4.5 GB
30000	14760 s	690.1 s	889.0 s	14.4 GB	10.1 GB
40000	8.4 h	0.44 h	1.13 h	25.6 GB	17.9 GB
50000	14.2 h	0.94 h	4.20 h	40.0 GB**	27.9 GB*
60000	27.3 h	/	8.61 h	57.6 GB**	/
70000	50.3 h	/	14.85 h	/	/
80000	91.3 h	/	40.90 h	/	/
90000	/	/	84.89 h	/	/
100000	/	/	127.91 h	/	/

Although the core innovation here is that OCMA can handle very large arrays in disk without the needs of large memory, OCMA’s memory-based algorithm by-itself is at least up-to-date with, if not faster than, state-of-the-art GRM analysis tools. Two of the most notable tools using GRM for association mapping and heritability analysis are GCTA (Yang *et al.* 2011) and EMMAX (Kang *et al.* 2010). EMMAX is not actively maintained and is orders of magnitude slower than OCMA. The most recent release of GCTA (1.9x.beta) is much faster and less resource intensive than its previous versions, but doesn’t provide a single function for only solving eigen-decomposition for the purpose of a comparison. However, the GREML (genomic-relatedness-based restricted maximum-likelihood) function that involves matrix inverse is a frequently used function estimating heritability etc. in genetic analyses, which provides a benchmark for comparison, albeit indirect. While the runtime and memory usage of GREML are higher than the use of matrix-factorization alone, we have derived formulas to use these results to estimate the actual use of resources (detailed and justified in the Supplementary Note II) in matrix-factorization. These estimates (Table 1, *N* = 10,000 to 50,000) show that OCMA is more efficient than GCTA when the memory is sufficient for GCTA to perform.

The above comparisons are based on the Linux version of OCMA. As mmap is a call at the level of operating system, the implementations of OCMA are quite different under different platforms. We have implemented the Mac OS and Windows version of OCMA. The performance of OCMA in the Windows system is presented in Table 3. The performance on Mac OS is similar to that on Linux. This is to be expected due to their shared Unix kernel. The performance on Windows is even faster than the Linux version, presumably due to the extra optimization by the operating system.

**Table 3:** Comparison of the runtime of OCMA under the Windows and Linux operating systems for eigen decomposition. The same machine described in the Table 1 and 2 was used. Four threads are used in the test. Time is measured by wall-clock (instead of CPU time). The sign “/” indicates that the system does not allow the calculation to happen due to limited memory and swap spaces

*N*	Windows		Linux	
	Memory	Disk	Memory	Disk
10000	46.2 s	46.2 s	41.6 s	55.0 s
20000	246.4 s	252.6 s	214.5 s	231.8 s
30000	760.7 s	775.3 s	690.1 s	889.0 s
40000	0.50 h	0.51 h	0.44 h	1.13 h
50000	1.02 h	1.05 h	0.94 h	4.20 h
60000	1.96 h	2.25 h	/	8.61 h
70000	/	3.43 h	/	14.85 h
80000	/	18.31 h	/	40.90 h
90000	/	52.53 h	/	84.89 h
100000	/	132.91 h	/	127.91 h

The performance of OCMA calculating SVDs (using the options “single” and “singular”) for very large genotype matrices (1 million simulated individuals times thousands of markers) is presented in Table 4. Evidently, the calculation of SVD is much faster and even less memory-intensive. Further, the performance of OCMA calculating a subset of SVs is displayed in Table 5. The runtime of calculating a small subset of SVs is not significantly faster than calculating all the SVs. This is because, for SVD computations, a significant proportion of runtime is spent on calculating the product of the matrix and its transpose (*i.e.*, A’ × A, where A is the *N* × *M* matrix). As this is a necessary step in the “sgesvdx” function of Intel MKL, calculating a subset of SVDs doesn’t save much time in this implementation.

**Table 4:** Runtime of OCMA for Singular Value Decomposition (SVD). Sample size *N* = 1 million. *M* is the number of selected genetic markers. The same machine described in the Tables 1 and 2 is used. Four threads are used in the test. Time is measured by wall-clock (instead of CPU time). The sign “/” indicates that the system does not allow the calculation to happen due to limited memory and swap spaces

*M*	Windows		Linux	
	Memory	Disk	Memory	Disk
1000	89.5 s	102.5 s	93.6 s	309.9 s
2000	226.1 s	230.9 s	208.0 s	3564 s
3000	450.8 s	523.4 s	391.8 s	11232 s
4000	0.41 h	0.42 h	/	4.94 h
5000	1.50 h	1.77 h	/	6.58 h
6000	3.21 h	3.38 h	/	9.94 h
7000	/	8.95 h	/	16.09 h
8000	/	16.40 h	/	21.16 h

**Table 5:** Runtime of OCMA for a subset of singular values. Sample size *N* = 1 million. *M*, the number of selected genetic markers, is 5,000. *K* is the number of top SVs. The same machine described in the Table 1 and 2 is used. Four threads are used in the test. Time is measured by wall-clock (instead of CPU time)

*K*	Windows		Linux	
	Memory	Disk	Memory	Disk
10	0.30 h	0.30 h	0.206 h	4.46 h
20	0.30 h	0.30 h	0.207 h	4.60 h
50	0.30 h	0.30 h	0.209 h	4.79 h
100	0.31 h	0.31 h	0.211 h	4.87 h
200	0.32 h	0.32 h	0.215 h	4.95 h
500	0.32 h	0.32 h	0.225 h	5.01 h
1000	0.33 h	0.33 h	0.230 h	5.10 h
2000	0.83 h	0.95 h	/	5.65 h

In addition to laptop/desktop-based data analyses, OCMA also supports HPC-based parallel computations that use many threads. We have tested OCMA on an HPC server and the results are presented in Table 6. As a comparison of the effectiveness of parallelization, we also have tested OpenBLAS, an optimized BLAS library using multi-thread. The same method, “ssyevd” in OpenBLAS is used for this test. Evidently, the parallelization in Intel MKL, which is adopted by OCMA, is more effective than the OpenBLAS alternative (Table 6).

**Table 6:** Comparison between OCMA and OpenBLAS, a multi-thread package for matrix factorization. The same function (“ssyevd”) in OpenBLAS is used. The computing node has two E5-2690V4 CPUs with 28 cores in total. The total memory available is 192GB. Environmental variables MKL_NUM_THREADS and OPENBLAS_NUM_THREADS are used to specify the number of threads in OCMA and OpenBLAS respectively

*N*	Memory	#Threads	Runtime	
			OCMA	OpenBLAS
10000	1.1GB	10	16.4 s	29.3 s
		20	14.3 s	24.3 s
20000	4.5GB	10	125.9 s	187.0 s
		20	73.1 s	156.5 s
50000	27.9GB	10	0.47 h	0.76 h
		20	0.25 h	0.52 h
100000	111.8GB	10	3.63 h	4.90 h
		20	1.90 h	3.54 h

As mmap involves complicated disk-memory data exchange, the memory usage of OCMA in practice is a dynamic function depending on the system status. However, based on the specification of Intel MKL and our implementation, we can provide a static analysis of the memory and disk usage. Quantitative analyses of the memory and disk usage of *mmap* are discussed and presented in Supplementary Tables 1-3. Intuitively there is an additional overhead incurred in the operating system, as the CPU must wait for data exchange between memory and disk. Under the desktop with Windows system, we monitored the CPU usage during the test and find this effect does exist but falls in a reasonable range. Supplementary Figures 1 and 2 show this effect for Eigen-decomposition and SVD respectively.

### Software Availability

The source code and executable are freely available at GitHub: https://github.com/precisionomics/OCMA. The repository provides OCMA for Windows (ocma-0.1.zip) and Linux/Unix systems (ocma-0.1.tar.gz). Upon decompression, one will see two folders: src and bin. The folder src contains all the source code, and the folder bin contains the compiled binary executable. Under the folder bin, two small matrices, SM10 and M3_4 for testing purpose are also provided.

The users can also build their own binary executable. The program is written in the C language, and it uses Intel MKL. Thus, in order to compile the source code, one needs to install C compiler, *e.g.*, the Intel C/C++ Compilers, the GNU C Compiler or Visual Studio C Compiler. Additionally, one needs to install Intel MKL. When C compiler and Intel MKL are available in the system, one can enter the src folder and modify the makefile based on the C compiler and the Intel MKL paths; then type make or nmake to compile. After that, the ocma executable will be generated in the bin folder.

## Discussion

OCMA allows researchers to factorize symmetric and non-symmetric of very large real-valued matrices, including GRMs, genotype matrices and any other real-valued matrices. Thus, OCMA enables many Big-Data applications that use distance and similarity matrices that do not use GRMs. Furthermore, the recent trend of analyzing multiple traits together in association mapping (Korte *et al.* 2012; Casale *et al.* 2015) and genomic selection (Tsuruta *et al.* 2011; Jia and Jannink 2012; Chen *et al.* 2016; 2017) involves matrices yet larger. The OCMA algorithm will be helpful in solving such larger matrices. Moreover, the study of Gene-Environment Interaction (GxE) in both health and agriculture also involves large matrices (Montesinos-López *et al.* 2016; Moore *et al.* 2018), which can be solved nimbly by OCMA.

As one may figure out from Figure S1, from the perspective of CPU usage, the swap within the operating system outperforms mmap for a matrix that is a little bit larger than the physical memory, especially in the case of calculating SVD. This might be because of the fact that the swap is supported by continuous and dedicated disk spaces designed to meet the specific requirements of memory virtualization. In contrast, mmap uses general file system that usually does not provide continuous space. So the mmap may consume more time than swap when the space for swap is sufficient. However, swap cannot meet the requirements of analyzing very large files. This is because (1) the performance of swap heavily relies on the setting of the operating system and how many other processes are sharing it; (2) swap requires administrators to set the parameters which is not available for the end users of an HPC; and (3) swap cannot be very large as it requires continuous and dedicated spaces in the disk. In contrast, mmap offers a scalable and flexible file mapping between the memory and the general file system, which does not suffer from the above limitations, therefore can be applied to very large files.

A trade-off in the design of OCMA is how to strike a good balance between space/speed and the nature of its memory visit. For instance, based on the Intel MKL website, it is recommended to use “ssyevr” for eigenvalue problems because “*its underlying algorithm is fast and uses less workspace*”. We have tested the function “ssyevr” against “ssyevd” (the function we adopted), and indeed we find that “ssyevr” uses around 65% memory that is required by “ssyevd”. However, “ssyevr” is slower when solving large matrices sized at the level of tens of thousands. In case of a very large matrix for which mmap is needed, the performance of “ssyevr” decreased dramatically. For instance, for a GRM of 80,000 that “ssyevd” can solve in 18.31 hr using a desktop with 24GB memory and 4 threads, “ssyevr” cannot be finished in 5 days. We expect this is because of the pattern of memory visit of “ssyevr”, which requires intensive workspace visits that needs more frequent data exchange between memory and disk. So although “ssyevr” needs less workspace (in terms of either memory or disk), its more frequent workspace visits leads to very poor performance in the context of an out-of-core implementation.

As an immediate future development, we plan to implement more factorization functions such as QR decomposition and Cholesky decomposition. Moreover, we will integrate OCMA with various tools especially the ones we have developed and maintained for years. For instance we will integrate OCMA with BGLR, a tool that we have developed for phenotype predictions using genomics data (de los Campos *et al.* 2013; Pérez and de los Campos 2014).

The initial release of OCMA is based on C language that is the default for *mmap*. We will develop APIs (Application Programming Interfaces) for R, Java and Python to accommodate broader communities. The extremely fast function in Intel MKL is optimized for Intel architectures that are supporting most current personal computers in the current market. Therefore, OCMA may be sub-optimal if running on other machines. However, other vendors, such as AMD, provide similar libraries. We are extending OCMA using the AMD Core Math Library, to cover the non-dominant architectures.

Another non-trivial extension will be the integration of OCMA with our existing tool JAWAMix5, a tool that enables out-of-core association mapping at the level of population genotype files (Long *et al.* 2013). The JAWAmix5 (Long *et al.* 2013) package uses HDF5 (https://www.hdfgroup.org) for accessing genotype data stored in disk. HDF5 builds indexes of genotype data to allow fast random access to data. JAWAmix5 offers scalable functions for the generation of GRM and association mappings using standard linear models as well as linear mixed models (Long *et al.* 2013). However, HDF5 is not efficient for complicated matrix operations because the index-based techniques are efficient only if the data are in a natural order and not frequently updated. So, JAWAmix5 loads matrices into the memory and conduct matrix factorization in memory. This was fine at that time (2013) however becomes increasingly a bottleneck when analyzing a sample with tens of thousands individuals. Although mmap is not as efficient as HDF5, it does allow users use memory mapping without concerning the properties of the composition of the underlying data.

HDF5 and mmap are two fundamentally different techniques. HDF5 facilitates a hierarchical indexing of the data so that they can be stored in the disk but randomly accessed by the computing processes as though they are in the main memory. This is convenient for some data that are naturally straightforward to index. However, if the reading and writing of the files cannot be well predicted, the HDF5-based solution could be very slow even if the file size is small. In contrast, mmap, despite its lower efficiency for well-formatted data (comparing with HDF5), offers a full “memory virtualization.” That is, one can just use disk as if it were memory regardless the type and organization of data, as well as how the users are going to read and write them. The operating system will handle which part of the data will be loaded and swapped. In statistical genomics, the genotype data (*e.g.*, a VCF file) meet the requirements of HDF5 perfectly because the genomic coordinate system offers a natural index and it is rarely updated during the statistical analyses. However, when there are many other intermediate quantities, *e.g.*, metrics to assist the decomposition of a matrix, for which one needs to generate and update frequently, the perfect choice may be mmap, which does not rely on the order of data. HDF5 and mmap nicely complement the advantages of each other. For the moment, based on JAWAmix5’s functions of association mapping and calculation of GRM and OCMA’s function of factorization of matrices, one can carry out GWAS using our out-of-core technologies. We plan to develop more functions for genomic predictions, *e.g.*, GBLUP and ssGBLUP (Aguilar *et al.* 2014) that have been extensively used in the fields.

## References

[bib1] Aguilar, I., I. Misztal, S. Tsuruta, A. Legarra, and H. Wang, 2014 PREGSF90 – POSTGSF90: Computational Tools for the Implementation of Single-step Genomic Selection and Genome-wide Association with Ungenotyped Individuals in BLUPF90 Programs. Proceedings of 10th World Congress of Genetics Applied to Livestock Production 2014: 680. 10.13140/2.1.4801.5045

[bib2] Buettner F. Natarajan K. N. Casale F. P. Proserpio V. Scialdone A., 2015 Computational analysis of cell-to-cell heterogeneity in single-cell RNA-sequencing data reveals hidden subpopulations of cells. Nat. Biotechnol. 33: 155–160. 10.1038/nbt.3102 25599176

[bib3] Casale F. P. Rakitsch B. Lippert C. Stegle O., 2015 Efficient set tests for the genetic analysis of correlated traits. Nat. Methods 12: 755–758. 10.1038/nmeth.3439 26076425

[bib4] Chen H. Iqbal M. Yang R.-C. Spaner D., 2016 Effect of Lr34/Yr18 on agronomic and quality traits in a spring wheat mapping population and implications for breeding. Mol. Breed. 36: 53 10.1007/s11032-016-0478-7

[bib5] Chen Y. Ren X. Zheng Y. Zhou X. Huang L., 2017 Genetic mapping of yield traits using RIL population derived from Fuchuan Dahuasheng and ICG6375 of peanut (*Arachis hypogaea* L.). Mol. Breed. 37: 17 10.1007/s11032-016-0587-3 28216998 PMC5285419

[bib6] Clark S. A. van der Werf J., 2013 Genomic best linear unbiased prediction (gBLUP) for the estimation of genomic breeding values. Methods Mol. Biol. 1019: 321–330. 10.1007/978-1-62703-447-0_13 23756897

[bib7] Collins R., 2012 What makes UK Biobank special? Lancet 379: 1173–1174. 10.1016/S0140-6736(12)60404-8 22463865

[bib8] de los Campos, G., and A. Grueneberg, 2017 A Suite of Packages for Analysis of Big Genomic Data [R package BGData version 1.0.0]. https://github.com/QuantGen/BGData 10.1534/g3.119.400018 PMC6505159 30894453

[bib9] de los Campos G. Hickey J. M. Pong-Wong R. Daetwyler H. D. Calus M. P. L., 2013 Whole-genome regression and prediction methods applied to plant and animal breeding. Genetics 193: 327–345. 10.1534/genetics.112.143313 22745228 PMC3567727

[bib10] de los Campos G. Sorensen D. Gianola D., 2015 Genomic heritability: What is it? PLoS Genet. 11: e1005048 10.1371/journal.pgen.1005048 25942577 PMC4420472

[bib11] Galinsky K. J. Bhatia G. Loh P.-R. Georgiev S. Mukherjee S., 2016 Fast principal-component analysis reveals convergent evolution of ADH1B in Europe and East Asia. Am. J. Hum. Genet. 98: 456–472. 10.1016/j.ajhg.2015.12.022 26924531 PMC4827102

[bib12] Jia Y. Jannink J.-L., 2012 Multiple-trait genomic selection methods increase genetic value prediction accuracy. Genetics 192: 1513–1522. 10.1534/genetics.112.144246 23086217 PMC3512156

[bib13] Kang H. M. Sul J. H. Service S. K. Zaitlen N. A. Kong S.-Y., 2010 Variance component model to account for sample structure in genome-wide association studies. Nat. Genet. 42: 348–354. 10.1038/ng.548 20208533 PMC3092069

[bib14] Kim H. Grueneberg A. Vazquez A. I. Hsu S. de los Campos G., 2017 Will big data close the missing heritability gap? Genetics 207: 1135–1145. 10.1534/genetics.117.300271 28893854 PMC5676235

[bib15] Koivula M. Strandén I. Su G. Mäntysaari E. A., 2012 Different methods to calculate genomic predictions–comparisons of BLUP at the single nucleotide polymorphism level (SNP-BLUP), BLUP at the individual level (G-BLUP), and the one-step approach (H-BLUP). J. Dairy Sci. 95: 4065–4073. 10.3168/jds.2011-4874 22720963

[bib16] Korte A. Vilhjalmsson B. J. Segura V. Platt A. Long Q., 2012 A mixed-model approach for genome-wide association studies of correlated traits in structured populations. Nat. Genet. 44: 1066–1071. 10.1038/ng.2376 22902788 PMC3432668

[bib17] Lin Z. Kahng M. Sabrin K. M. Chau D. H. P. Lee H., 2014 MMap: Fast billion-scale graph computation on a PC via memory mapping. Proc. IEEE Int. Conf. Big Data 2014: 159–164. 25866846 10.1109/BigData.2014.7004226 PMC4389765

[bib18] Lippert C. Listgarten J. Liu Y. Kadie C. M. Davidson R. I., 2011 FaST linear mixed models for genome-wide association studies. Nat. Methods 8: 833–835. 10.1038/nmeth.1681 21892150

[bib19] Listgarten J. Lippert C. Heckerman D., 2013 FaST-LMM-Select for addressing confounding from spatial structure and rare variants. Nat. Genet. 45: 470–471. 10.1038/ng.2620 23619783

[bib20] Listgarten J. Lippert C. Kadie C. M. Davidson R. I. Eskin E., 2012 Improved linear mixed models for genome-wide association studies. Nat. Methods 9: 525–526. 10.1038/nmeth.2037 22669648 PMC3597090

[bib21] Long Q. Zhang Q. Vilhjalmsson B. J. Forai P. Seren Ü., 2013 JAWAMix5: an out-of-core HDF5-based java implementation of whole-genome association studies using mixed models. Bioinformatics 29: 1220–1222. 10.1093/bioinformatics/btt122 23479353

[bib22] Lou T. P. Ludewigt B., 2015 MMAPDNG: A new, fast code backed by a memory-mapped database for simulating delayed γ-ray emission with MCNPX package. Comput. Phys. Commun. 194: 10–17. 10.1016/j.cpc.2015.04.005

[bib23] Masuda Y. Misztal I. Tsuruta S. Legarra A. Aguilar I., 2016 Implementation of genomic recursions in single-step genomic best linear unbiased predictor for US Holsteins with a large number of genotyped animals. J. Dairy Sci. 99: 1968–1974. 10.3168/jds.2015-10540 26805987

[bib24] McKusick M. K. Neville-Neil G. V. Watson R. N. M., 2014 The Design and Implementation of the FreeBSD Operating System, Addison-Wesley Professional.

[bib25] Montesinos-López O. A. Montesinos-López A. Crossa J. Toledo F. H. Pérez-Hernández O., 2016 A Genomic Bayesian Multi-trait and Multi-environment Model. G3 (Bethesda) 6: 2725–2744. 10.1534/g3.116.032359 27342738 PMC5015931

[bib26] Moore R. Casale F. P. Bonder M. J. Horta D. Consortium B., 2018 A linear mixed model approach to study multivariate gene-environment interactions. bioRxiv. 10.1101/270611 PMC6354905 30478441

[bib27] Peplow M., 2016 The 100,000 Genomes Project. BMJ 353: i1757 10.1136/bmj.i1757 27075170

[bib28] Pérez P. de los Campos G., 2014 Genome-wide regression and prediction with the BGLR statistical package. Genetics 198: 483–495. 10.1534/genetics.114.164442 25009151 PMC4196607

[bib29] Poulson J. Marker B. van de Geijn R. A. Hammond J. R. Romero N. A., 2013 Elemental: A new framework for distributed memory dense matrix computations. ACM Trans. Math. Softw. 39: 10.1145/2427023.2427030

[bib30] Rahmani E. Zaitlen N. Baran Y. Eng C. Hu D., 2016 Sparse PCA corrects for cell type heterogeneity in epigenome-wide association studies. Nat. Methods 13: 443–445. 10.1038/nmeth.3809 27018579 PMC5548182

[bib31] Ringnér M., 2008 What is principal component analysis? Nat. Biotechnol. 26: 303–304. 10.1038/nbt0308-303 18327243

[bib32] Salavert J. Tomás A. Medina I. Sadakane K. Blanquer I., 2016 Pair-end inexact mapping on hybrid GPU environments and out-of-core indexes. Curr. Bioinform. 11: 459–469. 10.2174/1574893611666160212235359

[bib33] Salavert J. Tomás A. Tárraga J. Medina I. Dopazo J., 2015 Fast inexact mapping using advanced tree exploration on backward search methods. BMC Bioinformatics 16: 18 10.1186/s12859-014-0438-3 25626517 PMC4384339

[bib34] Song N. Y. Son Y. Han H. Yeom H. Y., 2016 Efficient memory-mapped I/O on fast storage device. ACM Trans. Storage 12: 19 10.1145/2846100

[bib35] Speed D. Balding D. J., 2015 Relatedness in the post-genomic era: is it still useful? Nat. Rev. Genet. 16: 33–44. 10.1038/nrg3821 25404112

[bib36] Stegle O. Parts L. Piipari M. Winn J. Durbin R., 2012 Using probabilistic estimation of expression residuals (PEER) to obtain increased power and interpretability of gene expression analyses. Nat. Protoc. 7: 500–507. 10.1038/nprot.2011.457 22343431 PMC3398141

[bib37] Tsuruta S. Misztal I. Aguilar I. Lawlor T. J., 2011 Multiple-trait genomic evaluation of linear type traits using genomic and phenotypic data in US Holsteins. J. Dairy Sci. 94: 4198–4204. 10.3168/jds.2011-4256 21787955

[bib38] Van Essen B. Hsieh H. Ames S. Pearce R. Gokhale M., 2015 DI-MMAP—a scalable memory-map runtime for out-of-core data-intensive applications. Cluster Comput. 18: 15–28. 10.1007/s10586-013-0309-0

[bib39] Wang E. Zhang Q. Shen B. Zhang G. Lu X., 2014 Intel math kernel library, pp. 167–188 in High-Performance Computing on the Intel Xeon Phi, Springer International Publishing, Cham, Switzerland 10.1007/978-3-319-06486-4

[bib40] Wang L. Wu W. Xu Z. Xiao J. Yang Y., 2016 A high performance level-3 BLAS library for heterogeneous multi-GPU computing. Proceedings of the 2016 International Conference on Supercomputing 2016:20 ACM, New York, New York 10.1145/2925426.2926256

[bib41] Wang Z. Li J. Wu C. Yang D. Wang Z., 2015 HSPT: Practical implementation and efficient management of embedded shadow page tables for cross-ISA system virtual machines. Proceedings of the 11th ACM SIGPLAN/SIGOPS International Conference on Virtual Execution Environments. 2015: 53–64. 10.1145/2731186.2731188

[bib42] Yang J. Lee S. H. Goddard M. E. Visscher P. M., 2011 GCTA: a tool for genome-wide complex trait analysis. Am. J. Hum. Genet. 88: 76–82. 10.1016/j.ajhg.2010.11.011 21167468 PMC3014363

